# Metformin mitigates dementia risk among individuals with type 2 diabetes

**DOI:** 10.1186/s40842-024-00168-7

**Published:** 2024-05-10

**Authors:** Nicholas Aderinto, Gbolahan Olatunji, Emmanuel Kokori, Praise Fawehinmi, Abdulrahmon Moradeyo, Stephen Igwe, Rebecca Ojabo, Badrudeen Olalekan Alabi, Emmanuel Chuka Okafor, Damilola Ologbe, Ayobami Olafimihan, David B. Olawade

**Affiliations:** 1 Department of Medicine and Surgery, Ladoke Akintola University of Technology, Ogbomoso, Nigeria; 2 Department of Medicine and Surgery, University of Ilorin, Ilorin, Nigeria; 3 Southern Illinois University Edwardsville, Edwardsville, IL USA; 4 Afe Babalola University, Ado-Ekiti, Ado Nigeria; 5 William Harvey Hospital, Ashford, UK; 6 John H. Stroger Jr Hospital of Cook County, Chicago, IL USA; 7 Department of Allied and Public Health, School of Health, Sport and Bioscience, University of East London, London, UK

**Keywords:** Metformin, Dementia, Diabetes Mellitus, Cognitive impairment

## Abstract

This mini-narrative review explores the relationship between diabetes and dementia, focusing on the potential mitigating role of metformin in reducing cognitive decline among individuals with type 2 diabetes. The interplay of factors such as glycemic control, diabetic complications, and lifestyle influences characterises diabetes-related dementia. This review emphasises the significance of comprehensive diabetes management in addressing the heightened risk of dementia in this population. Methodologically, the review synthesises evidence from 23 studies retrieved through searches on PubMed, Embase, Google Scholar, and Scopus. Current evidence suggests a predominantly positive association between metformin use and a reduced risk of dementia in individuals with diabetes. However, the review shows the complex nature of these outcomes, revealing variations in results in some studies. These discrepancies show the importance of exploring dose–response relationships, long-term effects, and demographic diversity to unravel the complexities of metformin's impact on cognitive health. Limitations in the existing body of research, including methodological disparities and confounding variables, necessitate refined approaches in future studies. Large-scale prospective longitudinal studies and randomised controlled trials focusing specifically on cognitive effects are recommended. Propensity score matching and exploration of molecular mechanisms can enhance the validity of findings in clinical practice. From a clinical perspective, metformin can serve as a potential adjunctive therapy for individuals with diabetes at risk of cognitive decline.

## Introduction

Diabetes-related dementia is a significant concern due to the increased risk of dementia in individuals with type 2 diabetes [1]. The relationship between diabetes and dementia is complex and multifaceted [1]. Studies have shown that both low and high HbA1C levels are associated with an increased risk of dementia in individuals with diabetes, indicating a non-linear relationship [1, 2]. Additionally, uncontrolled diabetes has been linked to an elevated risk of Alzheimer's disease, highlighting the importance of glycemic control in mitigating dementia risk [3]. Furthermore, severe diabetic retinal disease has been identified as a potential risk factor for dementia in individuals with type 2 diabetes, emphasising the need for comprehensive management of diabetic complications to reduce the likelihood of developing dementia [4].

The impact of lifestyle factors on diabetes-related dementia has also been investigated, with studies suggesting that a combination of healthy lifestyle factors is associated with a reduced risk of dementia in patients with type 2 diabetes [5]. However, the aetiology of diabetes-related dementia remains unclear, and it has been proposed that dementia in diabetic patients should be regarded as an independent disease, distinct from Alzheimer's disease and vascular dementia, due to its unique pathophysiological characteristics related to diabetes [6–8].

The investigation into metformin as a potential mitigating agent for dementia risk among individuals with diabetes is grounded in the expanding body of evidence highlighting its plausible neuroprotective role [9]. Metformin's potential as a neuroprotective agent has been linked to its ability to lower mortality and age-related diseases independently of its impact on diabetes control [10–14]. Empirical evidence suggests that metformin might mitigate dementia risk by reducing oxidative stress, inflammation, and apoptosis and countering the deleterious effects of advanced glycosylation end products produced during hyperglycemia [10, 11]. These collective findings show metformin's potential not only in diabetes management but also in addressing neurological disorders. This study aims to review the current evidence for metformin as a mitigating agent for dementia risk among individuals with diabetes.

### Methodology

We searched PubMed, Embase, Google Scholar and Scopus to conduct this narrative review see Table 1. We formulated a database search strategy based on keywords such as "diabetes," "diabetes mellitus," "diabetes mellitus, Type 2", "metformin," "biguanides," "metformin benefits," "anti-diabetic medications," "memory," "cognition," "cognitive-impairment," "amnestic mild cognitive impairment," "Alzheimer's disease," "Parkinson's disease," and "dementia." We also used other texts selected based on the existing literature and/or obtained from related bibliographies, combined using Boolean operators as follows: ((dementia) OR (cognitive-impairment) OR (cognitive function) OR (neurodegenerative diseases)) AND ((metformin) OR (anti-diabetic drugs)). Furthermore, we manually searched relevant articles cited within the retrieved studies to avoid omitting important research articles.

**Table 1:** Methodology

**Methodology Component**	**Details**
**Database Search**	PubMed, Embase, Google Scholar, Scopus
**Search Strategy**	Formulated using keywords: "diabetes," "diabetes mellitus," "diabetes mellitus, Type 2," "metformin," "biguanides," "metformin benefits," "anti-diabetic medications," "memory," "cognition," "cognitive-impairment," "amnestic mild cognitive impairment," "Alzheimer's disease," "Parkinson's disease," and "dementia."
**Boolean Operators**	((dementia) OR (cognitive-impairment) OR (cognitive function) OR (neurodegenerative diseases)) AND ((metformin) OR (anti-diabetic drugs))
**Additional Sources**	Other texts from existing literature and related bibliographies
**Manual Search**	Reviewed relevant articles cited within retrieved studies
**Inclusion Criteria**	a) Results in English, b) Full text available, c) Specifically assessed dementia risk in patients with diabetes on metformin therapy
**Exclusion Criteria**	a) Missing data, b) Not focusing on metformin use in type 2 diabetes mellitus, c) Studies on patients with significant neurological, psychiatric disease or cancer, d) Studies in vitro or animal models
**Study Scope**	Randomised controlled trials, retrospective cohort studies, prospective observational studies, comparator studies, and case–control studies
**Excluded Types**	Books, letters, editorials, conferences, commentaries
**Data Extraction**	Evaluated study characteristics: Publication type, year, study design, study focus, sample size, number of positive and negative outcomes

We only considered articles that a) presented results in English, b) had full text available, and c) specifically assessed dementia risk in patients with diabetes who were on metformin therapy. On the other hand, we excluded studies with a) missing data, b) articles that did not focus on metformin use in type 2 diabetes mellitus, c) studies performed on patients with significant neurological, psychiatric disease or cancer, and d) studies performed in vitro or animal models. We limited the study scope to randomised controlled trials, retrospective cohort studies, prospective observational studies, comparator studies, and case–control studies but excluded books, letters, editorials, conferences, and commentaries.

During the data extraction process, we evaluated the study characteristics such as the publication type, year, study design, study focus, sample size, and the number of positive and negative outcomes. It is important to note that we focused on the probable benefit of metformin in mitigating dementia risk among individuals with diabetes despite the controversial nature of the topic.

### Current evidence in existing literature

Our review identified 23 studies, including sample sizes ranging from 305 to 446,105 participants see Table 2. A majority of these studies, 17 out of the 23 [10, 11, 13–27], reported positive outcomes regarding the relationship between metformin use and dementia risk in individuals with diabetes. Metformin is the preferred first-line drug for the treatment of type 2 diabetes mellitus [9]. It can be safely administered with other antidiabetic drugs and has been demonstrated to reduce insulin resistance and improve glycaemic control [9]. However, a review of clinical trials paints a mixed picture of the connection between the use of metformin and the incidence of dementia among patients with diabetes.

**Table 2:** Study characteristics

S/N	Study Name	Author names	Study design	Sample size	Positive Outcome	Negative Outcome
**1.**	Dementia Risk in Type 2 Diabetes Patients: Acarbose Use and Its Joint Effects with Metformin and Pioglitazone	Chin-Hsiao Tseng 2019 [20]	population-based retrospective cohort study	446,105	-Reduced risk of dementia associated with acarbose is observed in the female sex and in non-users of metformin -Users of all three drugs (acarbose, metformin, and pioglitazone) have the lowest risk of dementia	
**2.**	Long-Term Metformin Usage and Cognitive Function among Older Adults with Diabetes	Tze Pin Ng et al. 2014 [15]	population-based 4-year prospective study	365	-Long-term treatment with metformin may reduce the risk of cognitive decline in individuals with diabetes	No significant interactive effects of metformin use with APOE-4, depression, or fasting glucose level were observed
**3.**	Metformin use in elderly population with diabetes reduced the risk of dementia in a dose-dependent manner, based on the Korean NHIS-HEALS cohort	Yonghwan Kim et al. 2020 [19]	Longitudinal study	514,794	-The overall incidence rate of dementia was 11.3% (8.4% in men and 13.9% in women). Compared with metformin non-users, hazard ratios (95% confidence intervals) of low-, mid-, and high-users and non-diabetic individuals for dementia were 0.97 (0.73–1.28), 0.77 (0.58–1.01), 0.48 (0.35–0.67), and 0.98 (0.84– 1.15), respectively, in men, respectively, and 0.90 (0.65–0.98), 0.61 (0.50–0.76), 0.46 (0.36– 0.58), and 0.92 (0.81–1.04), respectively, in women, after full adjustment of confounding variables -Metformin use in an elderly population with DM reduced dementia risk in a dose–response manner	
**4.**	Metformin vs sulfonylurea use and risk of dementia in US veterans aged $65 years with diabetes	Ariela R. Orkaby et al. 2017 [13]	retrospective cohort study	432,922	-metformin was associated with a lower risk of subsequent dementia than sulfonylurea use in veterans,75 years of age - A lower risk of dementia was also seen in the subset of younger veterans who had HbA1C values $7% (HR 0.76; 95% CI 0.63–0.91), had good renal function (HR 0.86; 95% CI 0.76–0.97), and were white (HR 0.87; 95% CI 0.77–0.99)	
**5.**	Incidence of Dementia is Increased in Type 2 Diabetes and Reduced by the Use of Sulfonylureas and Metformin	Chih-Cheng Hsu et al. 2011 [11]	Representative cohort study	127,209	sulfonylureas and metformin may decrease the risk of dementia; together, these 2 OAs decrease the risk of dementia in T2DM patients by 35% over 8 years	
**6.**	Metformin and the Risk of Dementia in Type 2 Diabetes Patients	Chin-Hsiao Tseng 2019 [20]	retrospective cohort study	423,949	metformin use is associated with a reduced dementia risk	
**7.**	Metformin Use Is Associated With Slowed Cognitive Decline and Reduced Incident Dementia in Older Adults With Type 2 Diabetes: The Sydney Memory and Ageing Study	Katherine Samaras et al. 2020 [14]	prospective observational study	1,037	Older people with diabetes receiving metformin have slower cognitive decline and lower dementia risk	
**8.**	Effects of metformin exposure on neurodegenerative diseases in elderly patients with type 2 diabetes mellitus	Yi-Chun Kuan et al. 2017 [28]	Cohort study	Taiwan’s National Health Insurance Research Database > 50yrs 4651 metformin cohorts		-The metformin cohort had an increased risk of all-cause dementia (HR: 1.66, 95% CI = 1.35–2.04) - metformin exposure increased the risk of Alzheimer’s disease (HR: 2.13, 95% CI = 1.20–3.79) and vascular dementia (HR: 2.30, 95% CI = 1.25–4.22) - Long-term metformin exposure in patients with T2DM may lead to the development of NDs, including dementia and PD
**9.**	Metformin and Sulfonylurea Use and Risk of Incident Dementia	Jeffrey F. Scherrer et al. 2019 [21, 23]	cohort studies	75,187 VHA patients 10,866 KPW patients > 50yrs	Metformin use is associated with a modestly lower risk of incident dementia	
**10.**	The Correlation between Metformin Use and Incident Dementia in Patients with New-Onset Diabetes Mellitus: A Population-Based Study	Kuang-Hua Huang et al. 2023 [16]	population-based study	736,473	Patients with a low intensity of metformin use had a lower risk of dementia	higher doses of metformin with higher intensity exhibited no protective role in dementia
**11.**	The Association between Metformin Use and Risk of Developing Severe Dementia among AD Patients with Type 2 Diabetes	Ying xue et al. 2023 [29]	a propensity score-matched cohort	1306		metformin usage is not significantly associated with a decreased risk of severe dementia
**12.**	Metformin in Amnestic Mild Cognitive Impairment: Results of a Pilot Randomized Placebo Controlled Clinical Trial	Luchsinger, José A et al. 2016 [17]	Pilot Randomized Placebo Controlled Clinical Trial	80 55-90yrs	After adjusting for baseline ADAS-cog, changes in total recall of the SRT favored the metformin group (9.7 ± 8.5 versus 5.3 ± 8.5; *p* = 0.02)	7.5% of persons who did not tolerate metformin reported gastrointestinal symptoms
**13.**	Metformin, Lifestyle Intervention, and Cognition in the Diabetes Prevention Program Outcomes Study	José A. Luchsinger et al. 2017 [30]	observational study	2,280		-There were no differences in cognition across intervention arms -Type 2 diabetes was not related to cognition, but higher glycated hemoglobin at year 8 was related to worse cognition after confounder adjustment -Cumulative metformin exposure was not related to cognition
**14.**	Metformin intervention in obese non-diabetic patients with breast cancer: phase II randomized, double-blind, placebo-controlled trial	Kwang-Pil Ko et al. 2015 [22]	phase II randomized, double-blind, placebo-controlled trial	105	Metformin 1000 mg treatment had a favorable effect on controlling glucose and HbA1C levels in obese non-diabetic breast cancer patients, indicating prognostic importance	
**15.**	Antidiabetic medication and risk of dementia in patients with type 2 diabetes: a nested case–control study	Ida Kim Wium-Andersen et al. 2019 [10]	nested case–control study	176 250 patients	Use of metformin, DPP4 inhibitors, GLP1 analogs, and SGLT2 inhibitors were associated with lower odds of dementia after multiple adjustments (ORs of 0.94 (95% confidence interval (CI): 0.89–0.99), 0.80 (95% CI 0.74–0.88), 0.58 (95% CI: 0.50–0.67), and 0.58 (95% CI: 0.42–0.81), respectively	
**16.**	Association Between Metformin Initiation and Incident Dementia Among African American and White Veterans Health Administration Patients	Jeffrey F. Scherrer et al. 2019 [21, 23]	retrospective cohort study	73,761 > 50yrs	- metformin vs sulfonylurea use was associated with a significantly lower risk of dementia in African American patients (hazard ratio [HR] = 0.73; 95% CI, 0.6–0.89) but not white patients (HR = 0.96; 95% CI, 0.9–1.03) - Among those aged 65 to 74 years, metformin was significantly associated with lower risk of dementia in both races - Metformin was not associated with dementia in patients aged ≥ 75 years	
**17.**	Comparative effect of metformin versus sulfonylureas with dementia and Parkinson’s disease risk in US patients over 50 with type 2 diabetes mellitus	Danielle Newby et al 2022 [24]	comparator study	96 140 metformin users 16 451 sulfonylurea users	Metformin users compared with sulfonylurea users were associated with a lower risk of all-cause dementia, AD and VD but not with PD or MCI	
**18.**	Long term treatment with metformin in patients with type 2 diabetes and risk of vitamin B-12 deficiency: randomised placebo controlled trial	Jolien de Jager et al. 2010 [25]	Multicentre randomised placebo controlled trial	390	Long term treatment with metformin increases the risk of vitamin B-12 deficiency, which results in raised homocysteine concentrations	
**19.**	Collocation of metformin and dipeptidyl peptidase-4 inhibitor is associated with increased risk of diabetes-related vascular dementia: A single hospital study in Northern Taiwan	I-Shiang Tzeng et al. 2023 [26]	observational and data-driven studies	67,281	the combination therapy of metformin and DPP-4 inhibitor may increase the risk of dementia compared with that in the control group (adjusted hazard ratio, 1.11; 95% confidence interval, 1.06–1.15; *p* ≤ 0.001)	
**20.**	Extended-release metformin improves cognitive impairment in frail older women with hypertension and diabetes: preliminary results from the LEOPARDESS Study	Mone et al., 2023 [27]	Randomised Control Trial	145	Significant different cognitive performance compared to baseline in the group of frail women treated with extended-release metformin	
**21.**	Metformin attenuates LPS-induced neuronal injury and cognitive impairments by blocking NF-κB pathway	Zhou et al., 2021 [31]	Preclinical Study		Metformin administration alleviates the LPS-induced memory dysfunction and improves synaptic plasticity	
**22.**	Metformin restores cognitive dysfunction and histopathological deficits in an animal model of sporadic Alzheimer's disease	Rabieipoor et al., 2023 [32]	Preclinical Study		Metformin decreased inflammatory cells and reactive astrocytes as well as the dying neurons in the hippocampus region and the cortex in SAD, and improved the cognitive performance	
**23.**	Effect of Metformin on Short-Term High-Fat Diet-Induced Weight Gain and Anxiety-Like Behavior and the Gut Microbiota	Ji et al., 2019 [33]	Preclinical Model		For a short-term 3-week co-treatment, metformin alleviated the HFD-induced increase in body weight, the increase in adipocyte size and furthermore, the anxiety-like behavior	

The findings of observational studies examining the possible link between metformin and dementia risk have been inconclusive. Eleven (57.9%) of the 19 analysed publications had positive results, proving that metformin may help lower the risk of dementia [10, 11, 13, 14, 18–24, 27]. Five articles (26.3%) had an elevated risk [25, 26, 28–30], whereas three (15.8%) provided a condition for decreased risk [15–17]. A retrospective cohort study by Chin-Hsiao Tseng indicated a lower risk when metformin was used with other medications, such as acarbose and pioglitazone [18]. At the end of a 6-month follow-up study, a significant difference in cognitive performance compared to baseline in frail women treated with extended-release metformin (p: 0.007) was observed [27]. Huang et al. highlighted the protective benefits of metformin when used at a low dose [16]. At the same time, Huang et al. reported higher doses of metformin with a higher intensity showed no protective role against dementia [16]. However, cohort studies by Yi-Chun Kuan showed mixed results. They raised questions because they linked long-term metformin use to a higher risk of dementia from all causes, including vascular disease and Alzheimer's disease [28, 32]. Scherrer et al. showed that the effects of metformin vary in different subpopulations, indicating a lower risk in some individuals (> 50 years) [21].

Furthermore, the results from I-Shiang Tzeng raise questions about the possibility that metformin and DPP-4 inhibitor combination therapy alleviated the risk of dementia [26]. These varied results highlight the complex nature of the connection between dementia and metformin use and highlight the need for additional studies, especially examining dose–response interactions, long-term effects, and demographic diversity to offer a more thorough understanding. Among the notable findings is a study conducted by Chin-Hsiao Tseng in 2019, which indicated a reduction in the risk of dementia associated with metformin, particularly in the female population [18]. Furthermore, the use of a combination of three drugs (Metformin, acarbose, pioglitazone) was associated with the lowest risk of dementia, as highlighted in the same study [18]. Additionally, a study by Yonghwan Kim et al. demonstrated a dose–response relationship, revealing that Metformin use in an elderly population with diabetes mellitus contributed to a reduction in dementia risk [19]. However, a retrospective cohort study by Ariela R. Orkaby et al. in 2017 suggested that metformin was associated with a lower risk of subsequent dementia compared to sulfonylurea use in veterans aged 75 years and older [13]. Notably, a lower risk was also observed in a subset of younger veterans who maintained an HbA1C value of 7% and exhibited good renal function [13]. In the 2015 study by Kwang-pil Ko et al., a comprehensive evaluation of metformin's efficacy in modulating physical and mental profiles was undertaken, revealing favourable outcomes [22]. Specifically, within the age group of 65 to 74 years, metformin demonstrated a statistically significant association with a reduced risk of dementia across various racial categories. However, a distinctive pattern emerged among patients aged 75 years and older, as metformin exhibited no statistically significant association with dementia within this older demographic [23].

Theoretically, antidiabetic drugs designed to ameliorate insulin resistance within the brain hold promise in preventing Alzheimer's disease or dementia [18, 31]. In a study involving 17,200 new users of metformin, a lower risk of dementia was reported in a subset of younger veterans exhibiting HbA1C values ≥ 7%, those with good renal function, and individuals of white ethnicity [13]. In a study conducted, T2DM compared with no medication, sulfonylureas alone reduced the HR from 1 to 0.85 (0.71–1.01), metformin alone to 0.76 (0.58–0.98), while with combined oral therapy, the HR was 0.65 (0.56–0.74) [20]. Adjustments included cerebrovascular diseases so that non-stroke-related dementias were found to be decreased in DM with sulfonylurea and metformin therapy. T2DM increases the risk of dementia more than 2-fold.

Elevated blood glucose levels pose a potential threat to cerebral function, contributing to an elevated risk of dementia in individuals with diabetes [19, 31]. The link between diabetes and dementia is likely multifactorial, involving mechanisms such as inflammation, oxidative stress, atherosclerosis, amyloid-β deposition, brain insulin resistance accompanied by hyperinsulinemia, advanced glycation end-products (AGEs), and dysregulation of lipid metabolism [20, 33]. Metformin, recognised as the primary first-line therapy for type 2 diabetes mellitus, operates by curbing hepatic gluconeogenesis and augmenting muscular glucose uptake by activating 5'-adenosine monophosphate-activated protein kinase (AMPK) [21]. Beyond its glucose-lowering effects, metformin has demonstrated additional benefits in individuals with type 2 diabetes, including reducing the risk of atherosclerotic events, protection against certain cancers, and an anti-ageing effect [20].

The potential neuroprotective effects of metformin are suggested to stem from its capacity to inhibit inflammatory responses and enhance cognitive function [16]. Apolipoprotein E (APOE), a crucial protein in lipid transport and brain injury repair, is implicated in Alzheimer's disease risk [21]. Specific APOE gene polymorphisms, particularly the ε4 allele, elevate the risk of AD, while the ε2 allele is associated with reduced risk [10]. The APOE ε4 allele is also linked to an increased risk of cerebral amyloid angiopathy and age-related cognitive decline. A recent study hinted at an association between metformin use and a faster decline in delayed memory among carriers of the APOE ε4 allele, prompting the need for further research to elucidate the potential influence of APOE ε4 genotype on the therapeutic effects of metformin [29].

### Limitations and future directions

Existing studies on metformin’s involvement in reducing dementia risk in patients with diabetes have significant limitations that should be considered. First, many studies have methodological variances, such as differences in study design, sample size, and outcome measures. This variation makes obtaining standardised results difficult and direct comparisons between investigations difficult. Furthermore, the heterogeneity within the examined groups, which includes age and diabetes duration, complicates interpretation and restricts the generalizability of the findings. Most observational studies failed to address bias or did not address it clearly, making the evidence less efficient. Another significant issue is the possibility of confounding variables influencing the outcomes. Factors such as genetic predisposition, lifestyle decisions, and concurrent pharmaceutical use may all impact cognitive performance independent of metformin, making it difficult to assign observed effects to medication alone. Furthermore, contradictions in studies are exacerbated by differences in the definitions of dementia and cognitive decline between studies.

Future studies should target certain areas to address these constraints and to increase understanding. Large-scale, well-designed, prospective longitudinal studies with long follow-up periods can provide stronger data and aid in determining causation. In addition, randomised controlled trials (RCTs) focusing only on the cognitive effects of metformin would provide more control over confounding factors. Subgroup analyses within the diabetic population, considering variables such as age, sex, and diabetes management details, would help better understand the influence of metformin on various patient groups. Applying propensity score matching, or at the very least, a match for age, sex, and health status, will improve data validity by lowering baseline variability and, if possible, investigate the relationship between metformin usage, B-12 vitamin levels, and dementia. To inform clinical practice, it is critical to investigate dose–response relationships and optimal dosages for potential cognitive benefits.

Furthermore, a thorough examination of the molecular mechanisms underlying the influence of metformin on cognitive performance is required. This knowledge can guide focused therapies and identify individuals most benefit from metformin therapy. Future research should prioritise uniform study designs, investigate specific demographic subgroups, and explore molecular causes to improve the reliability and usefulness of the findings in clinical practice.

### Implications for clinical practice

**Fig. 1:**
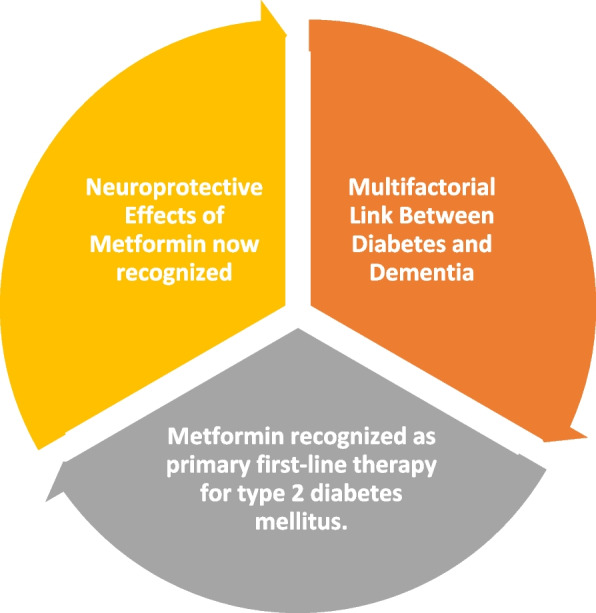
Metformin in dementia risk in type 2 diabetes

Clinically, the favourable results observed in multiple studies imply that metformin may be a feasible alternative for people with diabetes, particularly for those at risk of cognitive loss see Fig. 1.

Healthcare practitioners should inform patients about the potential cognitive benefits in addition to glycemic control. However, care is advised owing to inconsistent findings and potential issues, such as the variation in the metformin outcome, increased risk of vitamin B-12 insufficiency, and identified risk with certain combinations, emphasising the importance of tailored treatment programs and regular cognitive monitoring. A multidisciplinary approach that combines endocrinologists, neurologists, and senior experts is required to address the complicated connection between diabetes control and cognitive health. Senior experts such as diabetologists are key in tailoring diabetes treatment plans to achieve optimal glycemic control [34]. In addition, it is essential also to involve psychologists and occupational therapists. These professionals play pivotal roles in the identification, comprehensive assessment, and rehabilitation processes associated with dementia [35]. They collaborate closely to develop tailored interventions that address cognitive deficits and consider the individual's emotional and functional aspects [36]. This collaborative effort ensures a more personalised approach to patient care.

At the public health level, awareness programs should be launched to educate diabetic patients about the potential cognitive consequences of metformin and the significance of making informed decisions. Comprehensive studies investigating dose–response connections, long-term consequences, and population-specific effects should receive research funding. Public health guidelines must be revised to reflect increasing evidence, giving healthcare practitioners clear advice on using metformin in diabetes management taking both glycaemic control and cognitive outcomes into account. Policymakers should consider these findings when developing diabetes management policies and public health initiatives to ensure that possible cognitive effects are integrated into broader healthcare programs.

### Limitations and strengths of review

The review provides clear implications for clinical practice, suggesting that metformin may be a feasible adjunctive therapy for individuals with diabetes at risk of cognitive decline. The multidisciplinary approach recommended for navigating the complex relationship between diabetes control and cognitive health enhances the practicality of the review's recommendations. Also, the review identifies varied outcomes across studies, emphasising the complexity of the relationship between metformin use and dementia risk. This acknowledgement of diverse findings encourages a more cautious interpretation and highlights the need for further research. However, the included studies exhibit methodological disparities, including differences in study design, sample size, and outcome measures. This variation makes it challenging to obtain standardised results and directly compare findings between investigations.

## Conclusion

The body of evidence exploring metformin's role in mitigating dementia risk among individuals with diabetes presents a complex yet promising landscape. The interplay between diabetes and dementia shows the importance of glycemic control and comprehensive management of diabetic complications in reducing the likelihood of cognitive decline. This mini-narrative review reveals a spectrum of outcomes regarding the potential connection between metformin use and dementia risk in patients with diabetes. While a majority of studies suggest a positive association between metformin use and a reduced risk of dementia, the complex nature of these findings prompts a cautious interpretation. Dose–response interactions, long-term effects, and demographic diversity emerge as critical factors requiring further investigation to understand metformin's impact on cognitive health. Noteworthy variations in outcomes across studies highlight the need for standardised methodologies and robust study designs in future research endeavours.

## Data Availability

Data sharing is not applicable to this article as no datasets were generated or analysed during the current study.

## Abbreviations

AGEs: Advanced Glycosylation End Products
AD: Alzheimer's Disease
AMPK: 5'-Adenosine Monophosphate-Activated Protein Kinase
APOE: Apolipoprotein E
B-12: Vitamin B-12
ε2: Epsilon 2 (APOE gene polymorphism)
ε4: Epsilon 4 (APOE gene polymorphism)
HbA1C: Hemoglobin A1c
HR: Hazard Ratio
RCTs: Randomized Controlled Trials
T2DM: Type 2 Diabetes Mellitus
